# The power of a control qubit in weak measurements

**DOI:** 10.1038/s41598-017-05370-5

**Published:** 2017-07-25

**Authors:** Raul Coto, Víctor Montenegro, Vitalie Eremeev, Douglas Mundarain, Miguel Orszag

**Affiliations:** 1 Instituto de Fsíca, Pontificia Universidad Católica de Chile, Casilla, 306 Santiago, Chile; 2 Facultad de Ingeniería, Universidad Diego Portales, Av. Ejercito, 441 Santiago, Chile; 3 Department of Physics and Astronomy, University College London, London, WC1E 6BT UK; 4 Departamento de Fsíca, Universidad Católica del Norte, Casilla, 1280 Antofagasta, Chile; 5 Universidad Mayor, Santiago de Chile, Chile

## Abstract

In the late 80 s, a curious effect suggested by Aharanov *et al*. was found to lead to an anomalous amplification based on quantum measurements of weakly coupled systems. In this paper, we investigate the quantum control of the weak value amplification of a qubit system coupled to a meter. For the qubit system, the coupling occurs via a second non-interacting qubit, initially quantum correlated with the first one. We show that for weak measurements, the control can be remotely realized via the post-selected state of the second qubit or the degree of squeezing of the meter. In our study of the quantum control of the amplification, we can easily manipulate the degree of quantum correlations between the initially correlated qubits. We find that the degree of entanglement has no effect on the quantum control of the amplification. However, we also found a clear connection between the amplification and quantum discord like measurements as well as classical correlations between the qubits. Finally, we suggest an application of the amplification control on the enhancement of the quantum measurement accuracy, e.g. measuring the relative phase of the post-selected control qubit in a more precise way, as opposed to the non-amplified case.

## Introduction

Quantum Measurement Theory is as old as Quantum Mechanics. The collapse of quantum states in the measurement process, one of the basic assumptions in quantum mechanics and put forward by von Neumann in 1932^1^, strongly modifies such a state. The question then arises: what would happen if the interaction responsible for the measurement becomes weaker and weaker? For weak measurements (WM), a theory was developed by Aharonov and collaborators^2^, where the strong impact of the measurement is drastically reduced. It consists of a gradual accumulation of information during a finite interaction time of the meter and the system. As a matter of fact, the state is hardly changed, and after such a measurement the system is left in a state that in general is not an eigenstate of the observable under measurement, which seems to contradict the basic principles of Quantum Mechanics. However, this is not so, since the information obtained after one event is so modest, many measurement processes are necessary to actually obtain information on the system.

In the seminal paper^2^, Aharonov, Albert and Vaidman (AAV) showed that the combination of a weak measurement followed by a strong post-selection measurement may lead to some strange effects, usually referred to as an anomalous Weak Value Amplification (WVA), anomalous in the sense that the inferred mean value of the measured system variable lies outside its range of eigenvalues. The AAV results have been discussed in many papers^3–7^ and also experiments have been realized which have confirmed their predictions^8, 9^. More recently, ultra-sensitive measurements have been performed^10^ as well as precision metrology^11^ and an exciting experiment into the observation of the average trajectories of single photons in a two-slit interferometer^12^.

In parallel to weak measurements, the theory of quantum control of physical systems has been a central issue in recent quantum technology in relation to measurement-based processes^13–19^. For example, entangling mechanical motion to microwave radiation^20^, which is based on the fact that a measurement of one system can determine the state of the other. Furthermore, sometimes it is highly desirable to remotely control a specific process, bypassing restrictions related to the experimental setup, e.g. limited access to one of the subsystems.

In the framework of the AAV approach, this work proposes to clarify and resolve three research tasks related to the field of Quantum Information Science. The main task is devoted to the quantum control, in the processes of weak measurements, of a quantum system using *correlations* as resources. This control refers to remote manipulation of the amplification of the subsystem undergoing the AAV approach. The second task clarifies which kind of correlations are indispensable when the WVA occurs. Here, we find that Quantum Discord is a better resource than Entanglement and Classical Correlation, an outcome similarly observed in the context of other studies^21^. And, the third task deals with the problem of enhancing the amplification effect by squeezing the meter state, allowing one to extend the validity of the approximations made in ref. 2. In general, in this work we have used examples where the two qubits are in various states such as pure Bell, Werner or Bell diagonal states. On the other hand, we place the meter in a coherent, squeezed or infinitely wide Gaussian state in the p-representation. In the following we present our results in detail.

## Results

### Model of Weak Value Amplification assisted by entangled qubits

**Figure 1:**
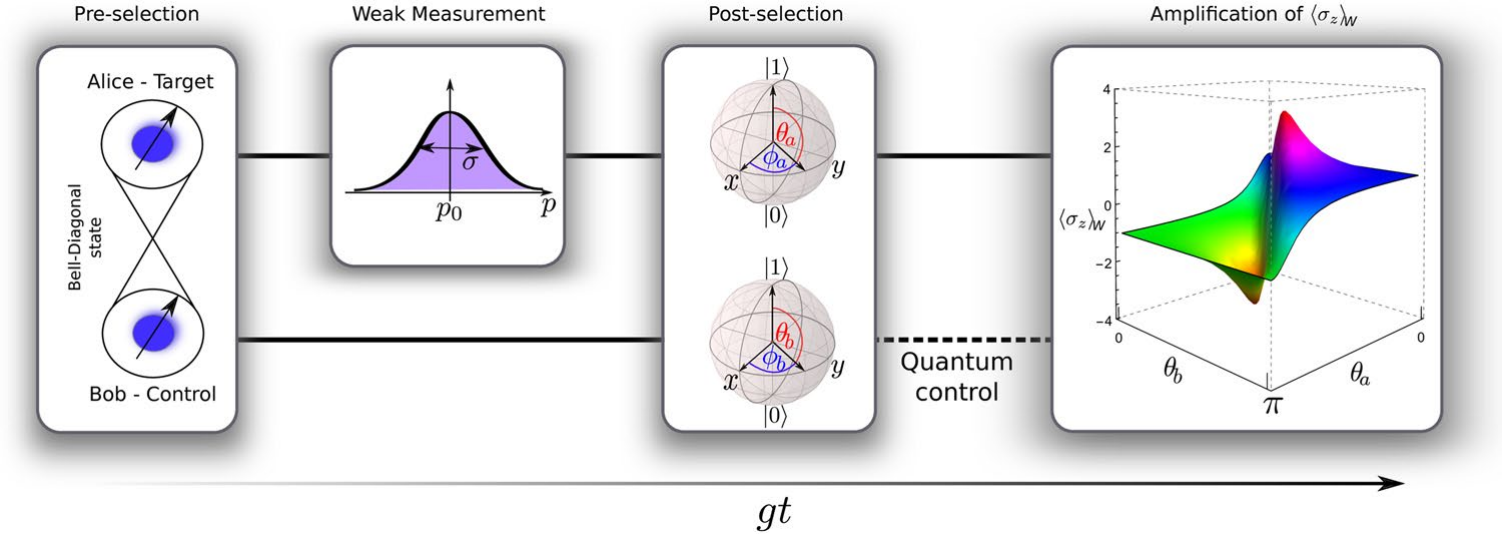
Model of weak measurement amplification assisted by quantum correlated qubits.

Let us consider two qubits (*a* and *b*), initially prepared in a Bell Diagonal (BD) state, *ρ* ^*Q*^, such that one of them (*a*) interacts dispersively with a meter, *ρ* ^*M*^. The second qubit (*b*) does not interact at all and is only linked to the system via the quantum correlations existing between the two qubits, see Fig. 1. The Hamiltonian in the interaction picture is

1 $$H=\hslash g{\sigma }_{3}^{a}x,$$

where *g* is the coupling strength between the qubit *a* and the meter; *σ*_3_ is the usual spin-1/2 Pauli operator in the *z*− direction and *x* denotes the continuous position of the meter. The initial state of the whole system, i.e. the two qubits together with the meter state is

2 $$\rho \mathrm{(0)}={\rho }^{Q}\otimes {\rho }^{M}=\frac{1}{4}({\mathbb{I}}+\sum _{j=1}^{3}{c}_{j}{\sigma }_{j}^{a}\otimes {\sigma }_{j}^{b})\otimes |\varphi \rangle \langle \varphi |,$$

where ${\mathbb{I}}$ is the identity operator in the two-qubit basis, *σ*_*j*_ are the Pauli operators and |*c*_*j*_| ≤ 1 are parameters satisfying the positivity of the density matrix. As known, the BD states are defined by a set of three parameters {*c*_1_, *c*_2_, *c*_3_} depicted in a three dimensional tetrahedron, a geometrical representation of the subsets of entangled, separable and classical states^22–24^.

From quantum measurement theory, the state of the meter must be expanded in the opposite conjugate variable appearing in Eq. (1), in our case this is the momentum subspace $|\varphi \rangle ={\mathrm{(2}\pi {\sigma }^{2})}^{-\mathrm{1/4}}{\int }_{-\infty }^{\infty }dp\,|p\rangle {e}^{-\frac{{(p-{p}_{0})}^{2}}{4{\sigma }^{2}}}$, where *σ* and *p*_0_ are the width and the center of the Gaussian profile respectively. Subsequently with respect to the time evolution, we proceed to post-select the target state using a generic qubit state in the Bloch sphere as $|{\psi }_{a}\rangle =\,\cos ({\theta }_{a}/2){|1\rangle }_{a}+\,\sin ({\theta }_{a}/2){e}^{i{\varphi }_{a}}{|0\rangle }_{a}$ (see Fig. 1). Notice that |1〉 and |0〉 are eigenstates of *σ*_3_ with eigenvalues 1 and −1 respectively. To calculate the post-selected state of the system ${\rho }_{{\psi }_{a}}=\langle {\psi }_{a}|\rho (t)|{\psi }_{a}\rangle$, we make use of the usual translational operator in quantum mechanics, *e* ^−*igtx*^|*p*〉 = |*p* − *gt*〉. Using the above equations and some algebra one gets

3 $$\begin{array}{rcl}{\rho }_{{\psi }_{a}} & = & \frac{1}{4\sigma \sqrt{2\pi }}\int dp\,dp^{\prime} \,{e}^{-\frac{{(p-{p}_{0})}^{2}}{4{\sigma }^{2}}-\frac{{(p^{\prime} -{p}_{0})}^{2}}{4{\sigma }^{2}}}\{{\cos }^{2}({\theta }_{a}/2){\rho }_{11}^{Q}|p-gt\rangle \langle p^{\prime} -gt|\\  &  & +\,{\sin }^{2}({\theta }_{a}/2){\rho }_{00}^{Q}|p+gt\rangle \langle p^{\prime} +gt|+\cos ({\theta }_{a}/2)\sin ({\theta }_{a}/2)\\  &  & \times [{\rho }_{10}^{Q}{e}^{-i{\varphi }_{a}}|p-gt\rangle \langle p^{\prime} +gt|+h.c.]\},\end{array}$$

with ${\rho }_{11}^{Q}={}_{a}\langle 1|{\rho }^{Q}{|1\rangle }_{a}={{\mathbb{I}}}^{b}+{c}_{3}{\sigma }_{3}^{b}$, ${\rho }_{00}^{Q}={}_{a}\langle 0|{\rho }^{Q}{|0\rangle }_{a}={{\mathbb{I}}}^{b}-{c}_{3}{\sigma }_{3}^{b}$, and ${\rho }_{10}^{Q}={}_{a}\langle 1|{\rho }^{Q}{|0\rangle }_{a}={c}_{1}{\sigma }_{1}^{b}-i{c}_{2}{\sigma }_{2}^{b}$, and ${{\mathbb{I}}}^{b}$ is the identity operator in the *b*-qubit basis.

According to Eq. (18) found in the Sec. Methods, one can easily observe that by measuring a meter variable one can indirectly evaluate the weak value of the system variable of interest. Because of this, after the post-selection, we are interested in the expectation value of the momentum, which can be found by tracing over the meter degrees of freedom. To investigate the effect of the control qubit on the amplification process, the momentum expectation value expression should remain as a function of the operators acting on the control qubit *b*. Furthermore, and importantly, since the control qubit *b* does not interact with the target qubit *a* nor with the meter, the specific time at which one acts on *b* will not affect the quantum dynamics.

Next, in order to calculate

4 $$\langle p\rangle \equiv \frac{{\langle T{r}_{M}({\rho }_{{\psi }_{a}}p)\rangle }_{b}}{{\langle T{r}_{M}({\rho }_{{\psi }_{a}})\rangle }_{b}}$$

we derive, after some simple algebra, an expression for $T{r}_{M}({\rho }_{{\psi }_{a}}p)$, which yields the following

5 $$\begin{array}{rcl}T{r}_{M}({\rho }_{{\psi }_{a}}p) & = & \frac{1}{4}[({{\mathbb{I}}}^{b}+{c}_{3}{\sigma }_{3}^{b}){\cos }^{2}({\theta }_{a}/2){K}_{11}+({{\mathbb{I}}}^{b}-{c}_{3}{\sigma }_{3}^{b}){\sin }^{2}({\theta }_{a}/2){K}_{00}\\  &  & +({c}_{1}{\sigma }_{1}^{b}\,\cos \,{\varphi }_{a}+{c}_{2}{\sigma }_{2}^{b}\,\sin \,{\varphi }_{a})\sin \,{\theta }_{a}{K}_{10}],\end{array}$$

where the integrals *K*_*ij*_, see Methods Eq. (22), are found to be *K*_11_ = *p*_0_ − *gt*, *K*_00_ = *p*_0_ + *gt*, ${K}_{10}={K}_{01}={p}_{0}{e}^{-{g}^{2}{t}^{2}\mathrm{/2}{\sigma }^{2}}$. The expression for $T{r}_{M}({\rho }_{{\psi }_{a}})$, and the denominator in Eq. (4) are calculated in a similar way. In fact, the expression is the same as above, found by simply replacing *K*_*ij*_ by *J*_*ij*_, with *J*_11_ = *J*_00_ = 1 and ${J}_{10}={J}_{01}={e}^{-{g}^{2}{t}^{2}\mathrm{/2}{\sigma }^{2}}$.

As mentioned above, one tries to understand the role of the control qubit *b* in the amplification process. To study this, let us consider two different approaches. (i) Firstly one traces over the control qubit *b*; (ii) Secondly one proceeds to perform a projection on the qubit *b*.

In the first case, considering Eq. (4), one gets $\langle p\rangle ={p}_{0}-gt\,\cos \,{\theta }_{a}$. It is easy to see that this WM value does not lead to any amplification (independent of the initial condition) and the expectation value of the momentum is bound by *p*_0_ ± *gt*. Furthermore, as previously known^25^, coherence plays a significant role in the weak amplification process, thus when tracing over the qubit *b*, one eliminates the coherence in qubit *a* and therefore the amplification effect is removed.

In the second approach, one projects the control qubit *b* to a similar state as that of the qubit *a*, i.e. $|{\psi }_{b}\rangle =\,\cos ({\theta }_{b}/2){|1\rangle }_{b}+\,\sin ({\theta }_{b}/2){e}^{i{\varphi }_{b}}{|0\rangle }_{b}$, it follows that by calculating as in ref. 2 the weak value for the spin operator, 〈*σ*_*z*_〉_*W*_ ≡ (*p*_0_ − 〈*p*〉)/*gt*, then by using the Eq. (21) in the Sec. Methods, the expectation value corresponds to

6 $${\langle {\sigma }_{z}\rangle }_{W}=\frac{{c}_{3}\,\cos \,{\theta }_{b}+\,\cos \,{\theta }_{a}}{1+{c}_{3}\,\cos \,{\theta }_{a}\,\cos \,{\theta }_{b}+{e}^{-\frac{{g}^{2}{t}^{2}}{2{\sigma }^{2}}}\,\sin \,{\theta }_{a}\,\sin \,{\theta }_{b}({c}_{1}\,\cos \,{\varphi }_{a}\,\cos \,{\varphi }_{b}+{c}_{2}\,\sin \,{\varphi }_{a}\,\sin \,{\varphi }_{b})}.$$

This is the principal analytical result of our work for the model of one control qubit in WM. In the following sections we analyze some particular cases such as Bell and Werner states, and thereafter the general BD states.

### Weak Value Amplification vs qubit correlations

**Figure 2:**
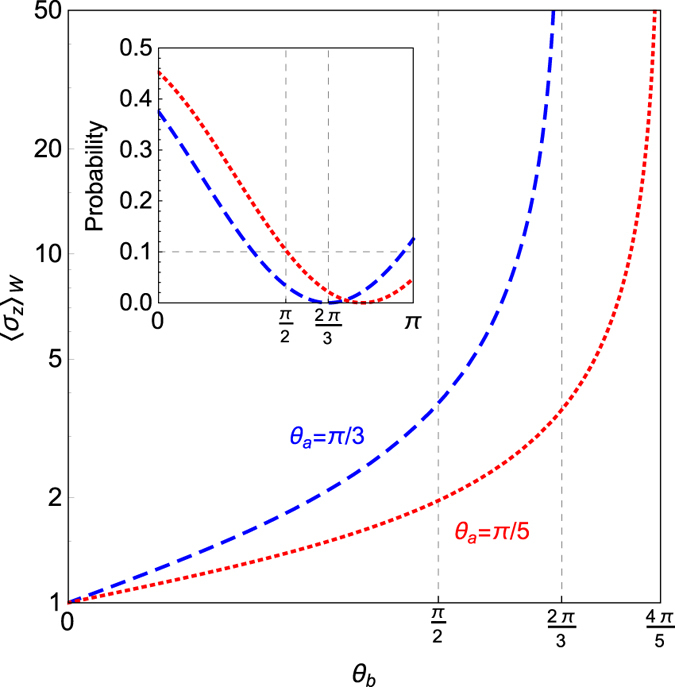
The weak value amplification for a *Bell* state, i.e. Eq. (7), managed by the projections of the target qubit *a*, and control qubit *b*, with a given probability (Inset). Here *δ* ≡ *ϕ*_*a*_ + *ϕ*_*b*_ = *π* and *σ* → ∞.

This section concerns the study of the amplification effect via WM and the quantum correlations shared by the qubits. The amplification effect in the AAV model appears when the denominator in the weak value tends to zero, i.e. when the pre- and post-selected states are almost orthogonal. Hence, as a simple and illustrative example let us consider the case of the two qubits initially prepared in a Bell state, e.g., $|{{\rm{\Phi }}}^{+}\rangle =(|{0}_{a}{0}_{b}\rangle +|{1}_{a}{1}_{b}\rangle )/\sqrt{2}$ (*c*_1_ = *c*_3_ = 1, *c*_2_ = −1) so Eq. (6) can then be reduced to

7 $${\langle {\sigma }_{z}\rangle }_{W}=\frac{\cos \,{\theta }_{b}+\,\cos \,{\theta }_{a}}{1+\,\cos \,{\theta }_{a}\,\cos \,{\theta }_{b}+{e}^{-\frac{{g}^{2}{t}^{2}}{2{\sigma }^{2}}}\,\sin \,{\theta }_{a}\,\sin \,{\theta }_{b}\,\cos \,\delta }$$

with *δ* = (*ϕ*_*a*_ + *ϕ*_*b*_). Now, as in ref. 2, if the meter state has a large Gaussian spread distribution over the momentum space, i.e. *σ* → ∞, one can easily check that there are several different combinations of the projection angles that allow us to make the denominator as small as required. For example, we consider the set of angles {*δ* = *π*, *θ*_*a*_ + *θ*_*b*_ = *π*} which leads to a large amplification with the constraint *θ*_*b*_ ≠ {0, *π*}. This constraint comes from the simple fact that for these *θ*_*b*_ values the coherence of qubit *a* disappears. The effect of WVA for the Bell state is represented in Fig. 2, alongside the computed probability of obtaining the WVA itself. The associated probability within the WM limit is calculated as |〈*ψ*_*i*_|*ψ*_*post*_〉|^2^, where |*ψ*_*i*_〉 is a Bell state |Φ^+^〉 and |*ψ*_*post*_〉 = |*ψ*_*a*_〉 ⊗ |*ψ*_*b*_〉, with |*ψ*_*a*_〉 and |*ψ*_*b*_〉 being the post selected states for qubits *a* and *b*. We observe a region where an important amplification takes place, where the probability is high enough from an experimental point of view. In Fig. 2 one finds that a twice amplified expectation value, exemplified by the red dotted line at *θ*_*b*_ = *π*/2, occurs with the probability ~10%.

To illustrate further the impact of the control qubit on the WVA, we proceed to measure the initial amount of quantum correlations between the qubits. To advance from simple to more elaborate scenarios, firstly we consider a *Werner* state, i.e. *ρ* ^*Q*^ ≡ *ρ*_*Werner*_ in Eq. (2). Werner states are a particular case of BD states which occur when *c*_1_ = *c*_2_ = *c*_3_ = −*c* and are defined as ref. 22 as ${\rho }_{Werner}=\mathrm{(1}-c){\mathbb{I}}\mathrm{/4}+c|{{\rm{\Psi }}}^{-}\rangle \langle {{\rm{\Psi }}}^{-}|$, where $|{{\rm{\Psi }}}^{-}\rangle =$ $(|{0}_{a}{1}_{b}\rangle -|{1}_{a}{0}_{b}\rangle )/\sqrt{2}$.

**Figure 3:**
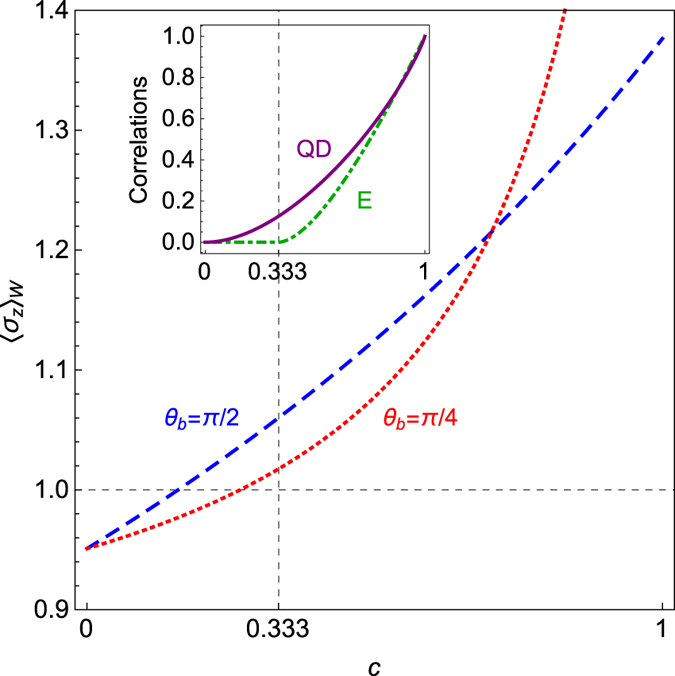
The weak value 〈*σ*_*z*_〉_*W*_ in Eq. (6) computed for a *Werner* state can be controlled by the projection of the control qubit *b* even for zero Entanglement (*E*) and non-zero Quantum Discord (*QD*) between the qubits (see Inset). The parameters are *θ*_*a*_ = *π*/10, *ϕ*_*a*_ = *ϕ*_*b*_ = 0 and *σ* → ∞.

Furthermore, it is known that Werner states exhibit entanglement if and only if *c* ≥ 1/3 (see Fig. 2 in ref. 22). Hence, it is clear that the Entanglement of Formation (*E*) vanishes for *c* < 1/3, while the Quantum Discord (*QD*) only vanishes at *c* = 0. Following the results above, we study the role of quantum correlations in the control of the two-qubit case. To achieve this, we show in Fig. 3 the amplification of the weak value given in Eq. (6) for the Werner state (*c*_*i*_ = −*c*). For this case, we have considered two different projections on the control qubit, *θ*_*b*_ = *π*/2 (blue dashed line) and *θ*_*b*_ = *π*/4 (red dotted line). Without loss of generality, for both cases we have fixed *ϕ*_*b*_ = *ϕ*_*a*_ = 0, *θ*_*a*_ = *π*/10. For *c* < 1/3, we observe the control of WVA with no entanglement, therefore, the entanglement does not play a relevant role in setting up the degree of quantum control. Thus, we find in this case that in order to have a control over the target qubit involved in the WVA, one must have a resource of quantum correlated states quantified by (in principle) Quantum Discord-like correlation measures rather than non-separability based on entanglement.

For completeness, let us consider the initial uncorrelated state (*QD* = 0), $|\phi \rangle =({|0\rangle }_{a}+{|1\rangle }_{a})/\sqrt{2}\otimes {|0\rangle }_{b}$. For this particular case and following the same procedure as before, it is straightforward to obtain the weak value:

8 $${\langle {\sigma }_{z}\rangle }_{W}=\frac{\cos \,{\theta }_{a}}{1+{e}^{-{g}^{2}{t}^{2}/2{\sigma }^{2}}\,\sin \,{\theta }_{a}\,\cos \,{\varphi }_{a}}.$$

One can see from this equation that the amplification of the mean value is achievable and it is not influenced by the control qubit state, meaning that tracing as well as projecting the quantum state gives the same result. As one would expect, this becomes a clear example of amplification of the target qubit, as in AAV theory, which depends on the weakness of the interaction and the post-selected state (*θ*_*a*_,*ϕ*_*a*_)^26^, but there is no control from the second qubit, since they are uncorrelated. In fact, one can find the amplification tending asymptotically to infinity when *θ*_*a*_ approaches *π*/2, with *ϕ*_*a*_ = *π*, although the associated probability goes to zero. To illustrate a fair comparison of our protocol in the WVA, we discuss the probability of success of amplifying 〈*σ*_*z*_〉_*W*_ as opposed to AAV’s original work (derived in Eq. (8)). For instance, we fix 〈*σ*_*z*_〉_*W*_ = 2, and find the optimized probability to be 12.5% versus 20%, for the two and one qubit cases respectively. As one would expect, the additional qubit deteriorates the probability of success, making the original protocol (AAV) the optimal one for amplification. Nevertheless, the obtained probability is high enough to exploit the advantages of the two-qubit setup for particular applications not accessible for the one qubit case, see Examples.

To find the weak interaction within the weak measurement framework, we proceed to set some routine values of the quantum dynamics, for instance, *gt* = *π* × 10^−3^, and *σ* = 1/2 (corresponding to a coherent state). These parameters give a quite accurate approximation of the case *σ* → ∞.

**Figure 4:**
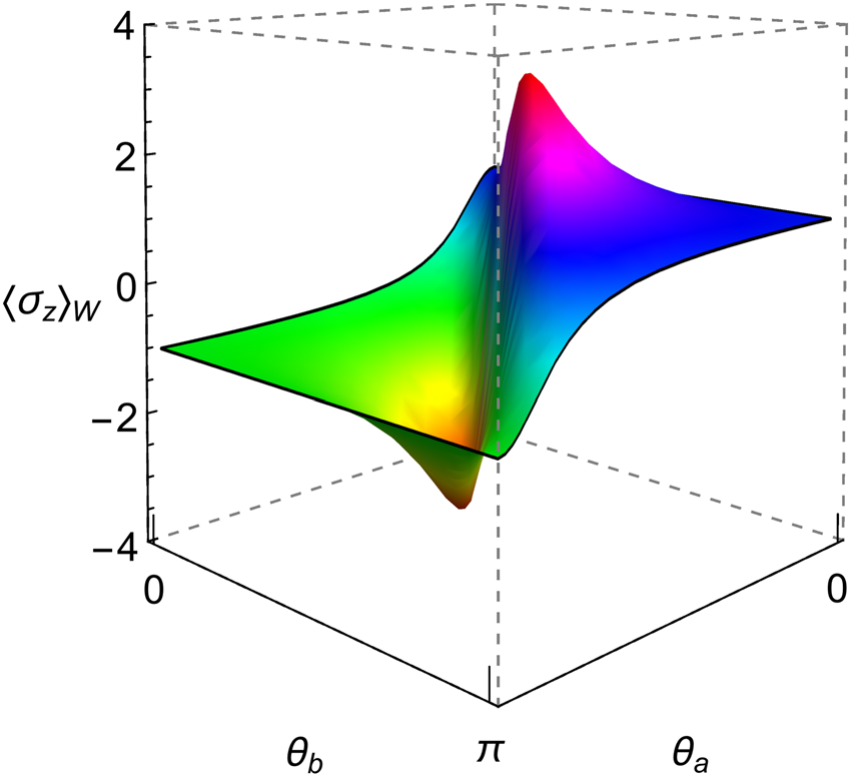
Weak value for an initial BD state with $\vec{c}=(-0.95,-0.95,-0.9)$ and varying the post-selection states for both qubits, e.g. the angles *θ*_*a*_ and *θ*_*b*_. Here *ϕ*_*a*_ = *ϕ*_*b*_ = *π*/4 and *σ* → ∞.

In Fig. 4 we have depicted the control of the WVA as a function of the pre- and post-selection parameters of both qubits. We have found that, by fixing an initial BD state (i.e. $\vec{c}$) and varying the angles *θ*_*a*_ and *θ*_*b*_ it is possible to optimize the amplification effect. Furthermore, the behavior of the Entanglement and the *QD* is similar to the previous Werner case (inset of Fig. 3). Therefore, for more general BD states, we have numerically confirmed that the Entanglement plays no role in the control of the WVA. Moreover, we point out some particular BD states, where the two qubits (target and control) share initially only classical correlations, like states with $\vec{c}=(\pm 1,0,0)$ and $\vec{c}=(0,\pm 1,0)$ ^23^, for which remote control over the WVA is possible. We present the details of this issue in the Discussion.

Considering the results of this section, we arrive to the following conclusions:

(i) Essentially, the remote quantum control consists of obtaining different WVA by manipulating the control qubit through the post-selected angles *θ*_*b*_ and *ϕ*_*b*_. This is the main result of our paper, since within the original work of AAV^2^ - where only one qubit is considered - the amplification depends only on the strength of the weak measurement, say the meter spread *σ*, and the pre and post-selected states. In our model, the control qubit *b* does not interact with the main target-meter system and actually it is only connected to the qubit *a* via the initial correlation. This suggests for the first time the idea that the WVA can be remotely switched on and off.
(ii) Further control might be achieved by conveniently choosing the initial BD state, i.e. control via the pre-selection of the two qubits and the quantum or classical correlations between them.

### Validation and limits of the approximations

At this stage, it is perhaps worth discussing whether the approximations made in the AAV theory are valid or not. However, within this work, we follow t he analysis in ref. 3, where for our particular case the conditions ensuring the validity of the previous calculations read:

9 $$\sigma \gg gt{\langle {\sigma }_{z}\rangle }_{W},$$

10 $$\sigma \gg gt{|\frac{\langle {\psi }_{post}|{\hat{{\bf{A}}}}^{n}{\rho }^{Q}|{\psi }_{post}\rangle }{\langle {\psi }_{post}|\hat{{\bf{A}}}{\rho }^{Q}|{\psi }_{post}\rangle }|}^{\frac{1}{n-1}},\quad n\ge 2.$$

As before, *σ* corresponds to the width of the Gaussian profile for the meter state, $\hat{{\bf{A}}}={\sigma }_{3}^{a}$ holds for the Pauli operator acting on the subspace of qubit *a* and |*ψ*_*post*_〉 = |*ψ*_*a*_〉 ⊗ |*ψ*_*b*_〉 is the final state of the qubits after the post-selection. Notably, and with some importance, the original restrictions shown in ref. 3 were obtained for an initial pure state, which is a particular case of the mixed state *ρ* ^*Q*^ (2) and it can be recast by replacing *ρ* ^*Q*^ = |*ψ*_*in*_〉〈*ψ*_*in*_|. Moreover, since we have ${\hat{{\bf{A}}}}^{n}={{\mathbb{I}}}^{a}$ for *n* even, and ${\hat{{\bf{A}}}}^{n}={\sigma }_{3}^{a}$ for *n* odd, then the restriction (10) can be split in two,

11 $$\sigma \gg gt\quad ,n-odd$$

12 $$\sigma \gg gt{|\displaystyle \frac{1/4(1+\sin {\theta }_{a}\sin {\theta }_{b}({c}_{1}\cos {\varphi }_{b}\cos {\varphi }_{a}+{c}_{2}\sin {\varphi }_{b}\sin {\varphi }_{a})+{c}_{3}\cos {\theta }_{a}\cos {\theta }_{b})}{1/4(\cos {\theta }_{a}+{c}_{3}\cos {\theta }_{b}+i\sin {\theta }_{a}\sin {\theta }_{b}(-{c}_{1}\cos {\varphi }_{b}\sin {\varphi }_{a}+{c}_{2}\sin {\varphi }_{b}\cos {\varphi }_{a})}|}^{\frac{1}{n-1}}\quad ,n-even.$$

One should note, we are only interested in cases where |〈*σ*_*z*_〉_*W*_| > 1 for which the condition (9) becomes a much stronger restriction than for (11). Now, we proceed to show whether the validity of AAV theory holds for each of the examples studied. First, we start with the initial state represented in Fig. 2, $|{{\rm{\Phi }}}^{+}\rangle =(|{0}_{a}{0}_{b}\rangle +||{1}_{a}{1}_{b}\rangle )/\sqrt{2}$ (*c*_1_ = *c*_3_ = 1, *c*_2_ = −1). It is easy to see that we fulfill the condition (9) everywhere, except for the two asymptotes, where the AAV approximation breaks down. The condition (12) has to be carefully studied, since the denominator may lead to divergences. For the case we are considering, *δ* = *ϕ*_*a*_ + *ϕ*_*b*_ = *π* and *θ*_*a*_ = *π*/3 (*θ*_*a*_ = *π*/5), the restriction reads,

13 $$\sigma \gg gt{|\frac{1+\cos ({\theta }_{b}+{\theta }_{a})}{\cos {\theta }_{a}+\cos {\theta }_{b}}|}^{\frac{1}{n-1}},$$

where at the critical point *θ*_*b*_ = 2*π*/3 (*θ*_*b*_ = 4*π*/5) the AAV is no longer valid and has been excluded from the analysis. Hence, the approximation is valid elsewhere. From Eq. (13), note that if *θ*_*a*_ is fixed, one can extend the validity by tuning *θ*_*b*_, i.e. we introduced another degree of freedom.

In a similar way, we checked the AAV theory for the Werner and Bell Diagonal states, for the set of parameters represented in Figs 3 and 4 respectively.

### Dynamical control of amplification with a squeezed meter state

Recently, it was pointed out that WVA benefits from a meter in a quantum state^27^. Therefore, we introduce another degree of quantum control by considering a squeezed meter state. Moreover, we show that via squeezing the meter state, we can extend further the validity of the theory to regions where for a coherent state of the meter the AAV theory fails.

**Figure 5:**
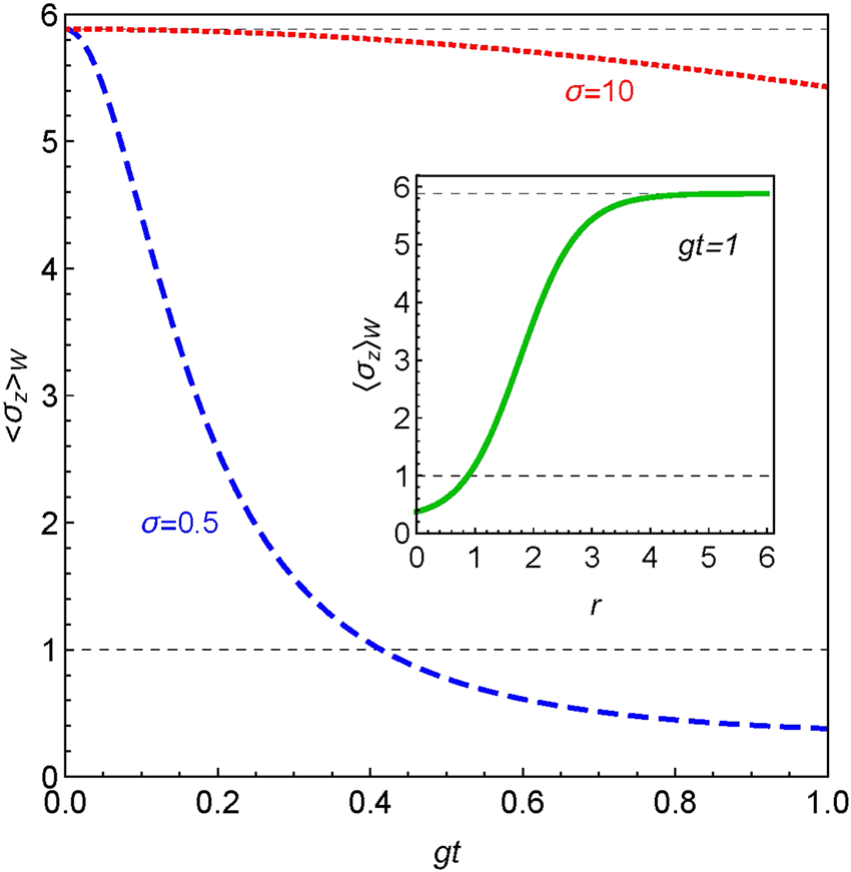
The weak value for a *Bell* state, i.e. Eq. (7), as function of the dimensionless time and *σ*. The momentum spread *σ* (the characteristic width of the meter device) sets a threshold for the validity of the AAV theory represented by the gridline. In the inset panel, we consider the case of a squeezed vacuum state, where *σ* is varied as a function of the squeezed parameter *r*. Here we define *δ* = *π* and *θ*_*a*_ = *θ*_*b*_ = 1.4 rad.

In Fig. 5, we show a case where the coupling constant *gt* was varied in relation to the weak interaction, for the two values of the momentum spread: where *σ* = 1/2 corresponds to a coherent state, while *σ* = 10 corresponds to a squeezed state. The upper gridline shows the validity of the AAV theory. One can easily see that for a coherent state, the approximation is only valid at the origin. However, for a squeezed state, we can extend the validity further. To show this more clearly, in the inset we plot 〈*σ*_*z*_〉_*W*_ as a function of the squeezing parameter *r* (*σ* ^2^ = *e* ^2*r*^/4), and one can observe that as we increase *r*, the amplification tends to the weak value of ref. 2.

### Is multiqubit control more efficient? Three qubits case

For further improvement of our proposal, one may consider adding more qubits for high quantum control. We will show that in fact this is not a beneficial procedure, by considering the case of only one control qubit (as the optimal scheme) we can show that the addition of another one only deteriorates the results obtained. Firstly, a direct generalization of the Bell state, |Φ^+^〉, as used within this paper, is the well-known Greenberger-Horne-Zeilinger (*GHZ*) state,

14 $${|{\rm{\Psi }}\rangle }_{GHZ}=(|000\rangle +|111\rangle )/\sqrt{2}$$

Then, one proceeds by fixing the parameters corresponding to the original qubits *a* (target) and *b* (first control qubit), say *θ*_*a*_, *θ*_*b*_ and *δ*, and by taking values that yield amplification with a finite probability. Subsequently, one varies the projection on the third qubit *e* (second control qubit). For a detailed derivation of the results, see the section Methods. We find numerically, that one can reach higher values for the amplification, but at the expense of having a lower probability than the previous one qubit control case. Therefore, such an effect does not lead to any improvement, and worse still, it compromises the experimental success.

Nevertheless, in connection to the three qubit scheme, there is more to say about the nature of the quantum correlations involved. It has been shown that the *GHZ* state (14) has only genuine three-partite correlations. While a *W* state, defined as

15 $${|{\rm{\Psi }}\rangle }_{W}=(|100\rangle +|010\rangle +|001\rangle )/\sqrt{3},$$

has multipartite correlations, e.g. pairwise Entanglement^28^. This means that for the state (14), when tracing out one of the qubits, the two remaining qubits are not quantum correlated. On the other hand, for (15), the opposite happens, and the remaining qubits are quantum correlated. To conclude this section, if one initially prepares the three qubits as a *GHZ* state, when tracing over one or two *control* qubits, the amplification is not possible. However, for the *W* state, when tracing over only one qubit, the amplification persists. This result suggests that the control of this type of amplification is intrinsically related to quantum correlations.

### Examples

**Figure 6:**
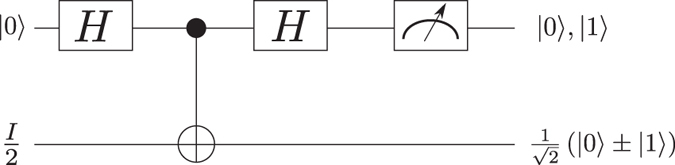
Gate sequence for generating coherence in Alice qubit, which leads to amplification. Without the postselection, Alice will remain in the mixed state and as a result, no amplification of any observable is possible.

Let us now examine two situations where the addition of the control qubit to the original setup of AAV becomes advantageous. Consider the initial condition where Alice (qubit A) is in a fully mixed state ${\mathbb{I}}\mathrm{/2}$ and Bob (qubit B) in a pure state |0〉. If Alice applies the AAV protocol she will not get any amplification due to the absence of coherence. Nevertheless, if Bob applies first a sequence of a Hadamard, C-NOT, Hadamard gates and finally post-selects the state |0〉 (|1〉), then Alice will end up in the state $\mathrm{1/}\sqrt{2}(|0\rangle +|1\rangle )$ ($\mathrm{1/}\sqrt{2}(|0\rangle -|1\rangle )$) and the amplification is possible. This sequence was inspired by ref. 29 and the scheme is sketched in Fig. 6.

Note that the control qubit allows us to enable the amplification, bypassing the need of initializing qubit A. To stress the utility of this remarkable tool and for the sake of explicitness, let us assume that Alice corresponds to a Carbon-13 nuclear spin in diamond which is coupled to the electronic spin of a nearby Nitrogen Vacancy colour centre (Bob) via hyperfine interaction. This nuclear spin is commonly in a mixed state due to the effect of thermal phonons, and its initialization can be a challenging task^30^. However, within our amplification scheme, one may consider the nuclear spin for amplification avoiding the technical challenge of its initialization.

The second example deals with the problem of getting more sensitivity from a qubit that is remotely connected to a WVA setup. As discussed previously, our amplification scheme relies on several quantum control degrees of freedom, being the projective post-selection measurements the most decisive ones to generate qubit WVA. Of course, one may question the feasibility of obtaining accuracy in the relative qubit phases^31, 32^, as well as its influence upon the final amplification. To address this issue and elucidate further, we draw attention to one particular but powerful application: enhancement of the control qubit measurement accuracy. In other words, we can rely on the weak value amplification protocol to gain further sensitivity on the post-selected phase *θ*_*b*_. To accomplish this, we make use of the sensitivity given by:

16 $$\eta =\frac{{\langle {\sigma }_{z}^{^{\prime} }\rangle }_{W}-{\langle {\sigma }_{z}^{0}\rangle }_{W}}{\partial /\partial {\theta }_{b}{\langle {\sigma }_{z}^{^{\prime} }\rangle }_{W}{|}_{{\theta }_{b}^{0}}},$$

where ${\langle {\sigma }_{z}^{\text{'}}\rangle }_{W}$, calculated using Eq. (7), is the output value measured for a phase *θ*_*b*_ assumed to be slightly displaced from ${\theta }_{b}^{0}=\pi \mathrm{/2}$. Needless to say this small deviation is something one would expect in any realistic experiment and ${\langle {\sigma }_{z}^{0}\rangle }_{W}$ is the theoretical prediction for a perfect measurement under ideal conditions. We now proceed to demonstrate that the amplification introduces a higher degree of accuracy. To this end, we evaluate *η* for two different phases measured on the target qubit, namely *ϕ*_*a*_ = *π* (amplification) and *ϕ*_*a*_ = *π*/2 (no amplification). We fixed the remaining parameters to be *ϕ*_*b*_ = 0, *θ*_*a*_ = *π*/3 and *σ* → ∞. Let us say that our meter, for example, cannot detect an angle variation of the output below 1%. Then, when there is no amplification (*ϕ*_*a*_ = *π*/2), the sensitivity is about 0.01. This means that angles below 0.01 rad cannot be resolved in the present configuration. However, when the amplification is switched on (*ϕ*_*a*_ = *π*) with a non-neglectable probability of ˜3.3%, the sensitivity corresponds to a value of 0.001. Thus, we can resolve angles up to 0.001 rad, being one order of magnitude smaller than the resolution with no amplification.

## Discussion

In the present work, we have studied the quantum control of the weak value amplification (WVA) of a qubit system coupled to a measurement device. On the one hand, a first qubit (target) is directly coupled to the detector device, whereas a second qubit (control) is linked to the former one solely via initial quantum correlations. Motivated by the non-local quantum control of the WVA, we have generalized the single qubit-meter system studied in ref. 2 towards an entangled multiqubit-meter scheme. We have shown that the two-qubit correlated scenario yields a success probability comparable with the original case of one qubit, but adds the missing feature of remote control. Moreover, the three qubit case only deteriorates the success rate, as opposed to the previous ones. Particularly, our theoretical analysis shows that quantum and classical correlations are important for the aforementioned control of the WVA. For some of the cases where the two qubits are initially classically correlated, the remote control over the WVA could occur. The explanation of these findings is based on the conclusions presented recently in ref. 25, where it was shown that the presence of coherence in the system is a necessary condition for the existence of WVA. Similarly, within our model the measurement of the control qubit *b* should generate coherence in qubit *a*, also observed in ref. 33. For the case of Bell Diagonal (BD) states with only classical correlations we find that the measurement of the qubit *b* generates the coherence in the qubit *a*, so WVA appears; in the case that the coherence is not generated, the WVA is not reported. On the other hand, for the BD states with quantum correlations (QD ≠ 0), the coherence is always generated in the system as result of the measurement of the control qubit, hence the protocol of WVA control is robust if the QD is present. From the two theorems given in Methods, we show that if for example, the asymmetric Discord vanishes in Alice subspace (target qubit), then there is an observable measured by Alice for which there is no WVA. On the other hand, if the discord is non-zero for *any* observable measured by Alice, there is *always* a post-selected state by Bob (control qubit) for which WVA is observed. This shows the role of the Quantum Discord in the amplification control.

Although Quantum Discord is a powerful resource for having remote control of the WVA, one also requires a projective set of individual local quantum operations on each qubit. For instance, for the pre-selected qubits in a general BD state it is possible to control the WVA via qubit projective post-selection measurements (see Fig. 4).

In the case of achieving WVA, besides the strongly controlled dependence of the amplification due to the phases (*θ*-azimuthal and *ϕ*-polar angles on the Bloch sphere) of the post-selected state of the control and target qubits, we also find the Gaussian spread of the meter state *σ* plays an important role. For instance, for a fixed coupling strength *gt* between the system and the meter, one can extend the validity of the AAV approximation by squeezing the meter state. This remark is in accordance with the original findings shown in ref. 2. In this case, to gather small amounts of information without perturbing the quantum state, the condition $gt\ll \sigma$ must be attained within the weak measurement framework - as we also require an approximation of the unitary evolution operator up to its first order in *gt*/*σ*. To illustrate this, we have explored different Gaussian spreads of the meter state by varying its degree of squeezing (see Inset of Fig. 5). We notice as we increase the squeezing parameter *r*, the approximation prevails and yields the exact weak value predicted by Aharonov *et*. *al*.

We believe that our present work suggests some interesting prospects for the development and implementation of a new set of experiments and technical tools related to ultra-small signal amplification, via remotely controlled weak measurements, by one or more correlated qubits.

## Methods

### Brief theory of weak measurements and weak values

Here we give a summary of the main results of the AAV’s standard approach^2^. One begins by preselecting the system S in an initial pure state, |*ψ*〉, such that the state of the system is given as |*ψ*〉 = ∑_*i*_ *α*_*i*_|*a*_*i*_〉, where {|*a*_*i*_〉} is the set of eigenstates of the system observable $\hat{{\bf{A}}}|{a}_{i}\rangle ={a}_{i}|{a}_{i}\rangle$.

On the other hand, if we let |*ϕ*〉 denote the wave function of the measurement apparatus or device detector, which is modeled in terms of the continuous variables $\hat{{\bf{X}}}$ and $\hat{{\bf{P}}}$, such that the initial detector state may be written as |*ϕ*〉 = ∫*ϕ*(*p*)*dp*|*p*〉, with $\varphi (p)={(2\pi {\sigma }^{2})}^{-1/4}{e}^{-{p}^{2}/4{\sigma }^{2}}$, where *σ* is a measure of the quantum fluctuations. In principle, one can define a WM as the limit when the standard deviation *σ* of the measurement outcome is much larger than the difference between the eigenvalues of the system. For strong measurements, the opposite is true.

The system-detector Hamiltonian, in the interaction picture, can be written as

17 $$\hat{{\bf{H}}}=g\hat{{\bf{A}}}\otimes \hat{{\bf{X}}},$$

where *g* is an interaction constant. Thus, the time evolution operator is $\hat{{\bf{U}}}(t)=\exp \{-\,i\frac{gt}{\hslash }\hat{{\bf{A}}}\otimes \hat{{\bf{X}}}\}$, where *t* is the interaction time. As a result, the global system-detector state after interaction is $|{\rm{\Psi }}\rangle =\exp \{-\,i\frac{gt}{\hslash }\hat{{\bf{A}}}\otimes \hat{{\bf{X}}}\}$ $|\psi \rangle \otimes |\varphi \rangle ={\sum }_{i}{\alpha }_{i}\int dp\,\varphi (p-gt{a}_{i})|p\rangle \otimes |{a}_{i}\rangle$.

If one takes the WM limit and post-selecting the system state |*ψ*_*post*_〉, the measurement device collapses to the state $|\varphi ^{\prime} \rangle =\exp (-\,i\frac{gt}{\hslash }{A}_{W}\hat{{\bf{X}}})|\varphi \rangle$, where *A*_*W*_ is the weak measurement value

18 $${A}_{W}=\frac{\langle {\psi }_{post}{\boldsymbol{|}}\hat{{\bf{A}}}{\boldsymbol{|}}\psi \rangle }{\langle {\psi }_{post}|\psi \rangle }\mathrm{.}$$

and the post-selection success probability is

19 $${P}_{post}={|\langle {\psi }_{post}|\psi \rangle |}^{2}.$$

For real *A*_*W*_^6^, it is easy to show that $|{A}_{W}|=\frac{\langle \varphi ^{\prime} |\hat{{\bf{P}}}|\varphi ^{\prime} \rangle }{gt}$, a quantity that in many cases has a value outside the range of the eigenvalues of the observable $\hat{{\bf{A}}}$, in particular in the limit 〈*ψ*_*post*_ *|ψ*〉 → 0. If, in general we write *A*_*W*_ ≡ *A* + *iB* as a complex number and let **M** be any pointer observable, one can easily prove that

20 $${\langle {\bf{M}}\rangle }_{f}={\langle {\bf{M}}\rangle }_{i}+igtA/\hslash {\langle \hat{{\bf{X}}}{\rm{M}}-{\rm{M}}\hat{{\bf{X}}}\rangle }_{i}+\frac{gtB}{\hslash }({\langle \hat{{\bf{X}}}{\rm{M}}+{\rm{M}}\hat{{\bf{X}}}\rangle }_{i}-2{\langle \hat{{\bf{X}}}\rangle }_{i}{\langle {\bf{M}}\rangle }_{i}),$$

with ${\langle {\bf{M}}\rangle }_{i}=\langle \varphi |\hat{{\bf{M}}}|\varphi \rangle /\langle \varphi |\varphi \rangle$, ${\langle {\bf{M}}\rangle }_{f}=\langle \varphi ^{\prime} |{\bf{M}}|\varphi ^{\prime} \rangle /\langle \varphi ^{\prime} |\varphi ^{\prime} \rangle$, where the *i* and *f* indices stand for the initial and final (post selection) states.

In particular, if *A*_*W*_ ≡ *iB* is purely imaginary, then ${\langle {\bf{X}}\rangle }_{f}={\langle {\bf{X}}\rangle }_{i}+2gtB/\hslash Var{({\bf{X}})}_{i}$. On the other hand, when *A*_*W*_ ≡ *A* is real

21 $${\langle {\bf{P}}\rangle }_{f}={\langle {\bf{P}}\rangle }_{i}-gtA$$

### Solving the integrals

${K}_{10}=\frac{1}{\sqrt{2\pi }\sigma }{\int }_{-\infty }^{\infty }dp(p-gt){e}^{-\frac{{(p-{p}_{0})}^{2}}{4{\sigma }^{2}}-\frac{{(p-{p}_{0}-2gt)}^{2}}{4{\sigma }^{2}}}$, by rearranging the exponential and using the substitution, *η* = *p* − *p*_0_, we get ${K}_{10}=\frac{1}{\sqrt{2\pi }\sigma }{e}^{-\frac{{g}^{2}{t}^{2}}{2{\sigma }^{2}}}{\int }_{-\infty }^{\infty }d\eta \,(\eta -gt+{p}_{0}){e}^{-\frac{{(\eta -gt)}^{2}}{2{\sigma }^{2}}}$.

Now we introduce a second variable *ξ* = *η* − *gt*, which leads to the result

22 $${K}_{10}={p}_{0}{e}^{-\frac{{g}^{2}{t}^{2}}{2{\sigma }^{2}}}$$

The remaining integrals *K*_*ij*_ and *J*_*ij*_ are calculated similarly.

### Weak measurements with many qubits

In this section we explore what happens if we include a third qubit *e* in the model, i.e. a second control. We are interested in two different types of tripartite quantum correlated initial states, namely the GHZ in Eq. (14) and “W”in Eq. (15). We initially focus on the GHZ initial state and we follow the same procedure as used to derive the numerator and denominator in Eq. (4), but written this time as a function of two control qubits, *b* and *e*, which give

23 $$\begin{array}{rcl}t{r}_{M}({\rho }_{{\psi }_{a}}) & = & \frac{1}{4}\{2{{\rm{\Pi }}}_{11}^{be}{\cos }^{2}({\theta }_{a}/2){J}_{11}+2{{\rm{\Pi }}}_{00}^{be}{\sin }^{2}({\theta }_{a}/2){J}_{00}\\  &  & +[{{\rm{\Pi }}}_{10}^{be}\,\sin \,{\theta }_{a}{e}^{i{\varphi }_{a}}{J}_{10}+h.c.]\},\end{array}$$

where ${{\rm{\Pi }}}_{ij}^{be}=|ii\rangle \langle jj|$ and *J*_*ij*_ are defined previously for Eq. (4). If one traces over one qubit and projects the other, or alternatively, one traces over both *b* and *e*, there will be no amplification. Nevertheless, projecting on both control qubits we find the denominator to be

24 $$\begin{array}{rcl}\langle {\psi }_{be}|t{r}_{M}({\rho }_{{\psi }_{a}})|{\psi }_{be}\rangle  & = & \frac{1}{16}\{8{\cos }^{2}({\theta }_{a}/2){\cos }^{2}({\theta }_{b}/2){\cos }^{2}({\theta }_{e}/2){J}_{11}\\  &  & +8{\sin }^{2}({\theta }_{a}/2){\sin }^{2}({\theta }_{b}/2){\sin }^{2}({\theta }_{e}/2){J}_{00}\\  &  & +[\,\sin \,{\theta }_{a}\,\sin \,{\theta }_{b}\,\sin \,{\theta }_{e}{e}^{i{\varphi }_{abe}}{J}_{10}+h.c.]\}\end{array}$$

where *ϕ*_*abe*_ = *ϕ*_*a*_ + *ϕ*_*b*_ + *ϕ*_*e*_. One can see that for the weak regime (*σ* → ∞) the solution {*θ*_*a*_ = *θ*_*b*_ = *θ*_*e*_ = *π*/2,*ϕ*_*a*_ = *ϕ*_*b*_ = *ϕ*_*e*_ = *π*} leads to amplification (where the denominator is zero). However, for the strong regime (*σ* → 0) will not yield any amplification, as pointed out previously in ref. 2 for only one qubit.

For the *W* initial state Eq. (15) the denominator reads

25 $$\begin{array}{rcl}t{r}_{M}({\rho }_{{\psi }_{a}}) & = & \frac{1}{6}\{2{{\rm{\Pi }}}_{0000}^{be}{\cos }^{2}({\theta }_{a}/2){J}_{11}\\  &  & +2({{\rm{\Pi }}}_{0101}^{be}+{{\rm{\Pi }}}_{1010}^{be}+{{\rm{\Pi }}}_{0110}^{be}+{{\rm{\Pi }}}_{1001}^{be}){\sin }^{2}({\theta }_{a}/2){J}_{00}\\  &  & +[({{\rm{\Pi }}}_{0001}^{be}+{{\rm{\Pi }}}_{0010}^{be})\sin \,{\theta }_{a}{e}^{i{\varphi }_{a}}{J}_{10}+h.c.]\},\end{array}$$

with ${{\rm{\Pi }}}_{ijkl}^{be}=|ij\rangle \langle kl|$. Once again, as we have found throughout this work, when tracing over the two control qubits, the amplification is annihilated, since the denominator is $1+{\sin }^{2}({\theta }_{a}/2)\ge 1$ projecting on *e*, one gets

26 $$\begin{array}{rcl}\langle {\psi }_{e}|t{r}_{b}[t{r}_{M}({\rho }_{{\psi }_{a}})]|{\psi }_{e}\rangle  & = & \frac{1}{6}\{2{\cos }^{2}({\theta }_{a}/2){\sin }^{2}({\theta }_{e}/2){J}_{11}+2{\sin }^{2}({\theta }_{a}/2){J}_{00}\\  &  & +\,\sin \,{\theta }_{a}\,\sin \,{\theta }_{e}\,\cos ({\varphi }_{a}-{\varphi }_{e}){J}_{10}\}\end{array}$$

In the second case above, the denominator does not vanish, but there is still an interference term: $\sin ({\theta }_{a})\,\sin ({\theta }_{e})\,\cos ({\varphi }_{a}-{\varphi }_{e})/2$, which amplifies the expectation value of momentum, i.e. $|\langle p\rangle -{p}_{0}|/gt\,\lessapprox \,4$. Therefore, for the initial *W* state given in Eq. (15), one can still find amplification after tracing over one of the qubits, unlike the GHZ case.

These important differences are related to the quantum correlations, as it is well known for the *GHZ* state that after tracing over one of three qubits, all the correlations between them are lost, since the *Quantum Correlations* are purely tripartite. However, for the *W* state the *Quantum Correlations* remain after tracing over one qubit, which is the reason behind the amplification of the momentum.

### On the importance of the quantum discord in the remote control of WVA

#### **Theorem 1.**

*If the asymmetric discord vanishes in Alice’s subspace, then there is an observable (or a complete set of eigenstates) measured by Alice, for which there is no weak value amplification, independent of the post-selected state (Bob)*.

*Proof*. Let us assume that the state is classical-quantum, that has the following structure:

$$\rho =p|{\varphi }_{a}\rangle \langle {\varphi }_{a}|\otimes |{\psi }_{1}\rangle \langle {\psi }_{1}|+(1-p)|{\varphi }_{a}^{\perp }\rangle \langle {\varphi }_{a}^{\perp }|\otimes |{\psi }_{2}\rangle \langle {\psi }_{2}|,$$

where $|{\varphi }_{a}\rangle ,|{\varphi }_{a}^{\perp }\rangle$ are orthogonal in Alice’s subspace. If |*ψ*_1_〉, |*ψ*_2_〉 are also orthogonal in Bob’s subspace, we have a strictly classical state, but in general it (or they) need not be.

If Bob selects a state |*ψ*_*b*_〉, Alice collapses to the state

$${\rho }_{a}=\lambda |{\varphi }_{a}\rangle \langle {\varphi }_{a}|+(1-\lambda )|{\varphi }_{a}^{\perp }\rangle \langle {\varphi }_{a}^{\perp }|,$$

with

$$\lambda =\frac{p|\langle {\psi }_{b}|{\psi }_{1}\rangle {|}^{2}}{p{|\langle {\psi }_{b}|{\psi }_{1}\rangle |}^{2}+\mathrm{(1}-p){|\langle {\psi }_{b}|{\psi }_{2}\rangle |}^{2}}\mathrm{.}$$

Hence, if Alice measures an observable with eigenstates |*ϕ*_*a*_〉 and $|{\varphi }_{a}^{\perp }\rangle$, there will be no amplification, since *ρ*_*a*_ is diagonal, that is, with no coherences, and independent of the post-selected state chosen by Bob.☐

#### **Theorem 2.**

*If the asymmetric discord is non-zero in Alice’s subspace, then for any observable measured by Alice, there is always a state post-selected by Bob such that WVA will be observed. (That is, the final ρ*_*a*_ *after post-selection will have non-zero coherences)*.

*Proof*. We can easily _*easily*_ prove the opposite.

If there is an observable A (or a set of eigenstates of A) for which Alice’s final state (after post-selection) is always diagonal in the basis of such an observable, independent of the post-selected state (Bob), then the state has zero discord in Alice’s subspace.

If the final state of Alice is always diagonal, for any post-selected state |*ψ*_*b*_〉, then it should have the following structure

$$\begin{array}{rcl}{\rho }_{ab} & = & {\rho }_{a}^{(1)}\otimes |{\psi }_{b}\rangle \langle {\psi }_{b}|+{\rho }_{a}^{(2)}\otimes |{\psi }_{b}^{\perp }\rangle \langle {\psi }_{b}^{\perp }|\\  &  & +\{{\rho }_{a}^{(3)}+{\alpha }_{1}|{\varphi }_{a}\rangle \langle {\varphi }_{a}^{\perp }|+{\beta }_{1}|{\varphi }_{a}^{\perp }\rangle \langle {\varphi }_{a}|\}\otimes |{\psi }_{b}\rangle \langle {\psi }_{b}^{\perp }|\\  &  & +\{{\rho }_{a}^{(4)}+{\alpha }_{2}|{\varphi }_{a}\rangle \langle {\varphi }_{a}^{\perp }|+{\beta }_{2}|{\varphi }_{a}^{\perp }\rangle \langle {\varphi }_{a}|\}\otimes |{\psi }_{b}^{\perp }\rangle \langle {\psi }_{b}|,\end{array}$$

where the ${\rho }_{a}^{(i)},i=1,2,3,4$ are diagonal terms in the $\{|{\varphi }_{a}\rangle ,|{\varphi }_{a}^{\perp }\rangle \}$ basis

$${\rho }_{a}^{(i)}={\lambda }_{i}|{\varphi }_{a}\rangle \langle {\varphi }_{a}|+{\lambda }_{i}^{\perp }|{\varphi }_{a}^{\perp }\rangle \langle {\varphi }_{a}^{\perp }|,i=1,\,\ldots \,4$$

The above structure guarantees that Alice’s state is diagonal after post-selecting |*ψ*_*b*_〉 or $|{\psi }_{b}^{\perp }\rangle$.

Now, if we assume that

$$|{\psi }_{Bob}\rangle =\alpha |{\psi }_{b}\rangle +\beta |{\psi }_{b}^{\perp }\rangle ,$$

then the conditions under which the state of Alice remains diagonal are easily obtained as

$$\begin{array}{rcl}{\alpha }^{\ast }\beta {\alpha }_{1}+{\beta }^{\ast }\alpha {\alpha }_{2} & = & 0,\\ {\alpha }^{\ast }\beta {\beta }_{1}+{\beta }^{\ast }\alpha {\beta }_{2} & = & 0.\end{array}$$

If we choose $\alpha =\beta =\frac{1}{\sqrt{2}}$, we get *α*_1_ + *α*_2_ = 0. On the other hand, if $\alpha =-i\beta =\frac{1}{\sqrt{2}}$, then *α*_1_ − *α*_2_ = 0. Since Alice’s final state should be diagonal for any post-selected state, it necessarily implies *α*_1_ = *α*_2_ = 0 and similarly *β*_1_ = *β*_2_ = 0.

Thus, the initial state has to be of the form:

$${\rho }_{ab}=|{\varphi }_{a}\rangle \langle {\varphi }_{a}|\otimes {\rho }_{b}^{(1)}+|{\varphi }_{a}^{\perp }\rangle \langle {\varphi }_{a}^{\perp }|\otimes {\rho }_{b}^{(2)},$$

which is a classical-quantum state with zero discord in Alice’s subspace.

In conclusion, if the state *ρ*_*ab*_ has non-zero quantum discord in Alice’s subspace, for any observable measured by Alice, then there will always be a post-selected state (Bob) that will generate WVA. ☐
